# Supplementary material for: Impact of Processing Methods on the In Vitro Protein Digestibility and DIAAS of Various Foods Produced by Millet, Highland Barley and Buckwheat
Source: Foods. 2023 Apr 20;12(8):1714. doi: 10.3390/foods12081714 (PMC10137793; doi:10.3390/foods12081714)
Supplement: Supplementary file 1 [file foods-12-01714-s001.zip › foods-2294855-supplementary.pdf]

Table S1

Amino acid (AA) compositions of raw grains, protein isolates (PI) and digest from cereal-based foods produced using grains (DPG) or flours (DPF) of millet, highland barley and buckwheat (g/100 g protein).

AA	Millet				Highland barley				Buckwheat			
	Raw grains	PI	DPG	DPF	Raw grains	PI	DPG	DPF	Raw grains	PI	DPG	DPF
Indispensable AA												
His	2.05±0.08^{b}	1.30±0.04^{d}	2.40±0.14^{a}	1.79±0.06^{c}	1.74±0.13^{b}	1.80±0.12^{b}	1.68±0.08^{b}	2.47±0.19^{a}	2.21±0.13^{b}	1.94±0.20^{b}	2.17±0.11^{b}	2.64±0.13^{a}
Thr	3.38±0.09^{bc}	3.03±0.06^{c}	4.32±0.26^{a}	3.60±0.14^{b}	2.89±0.14^{c}	3.01±0.09^{c}	3.97±0.19^{b}	4.77±0.08^{a}	4.65±0.10^{b}	4.64±0.18^{b}	4.36±0.19^{b}	5.17±0.15^{a}
Tyr	3.66±0.09^{c}	4.33±0.22^{b}	4.89±0.23^{a}	4.52±0.04^{ab}	4.03±0.15^{c}	4.92±0.07^{b}	4.92±0.34^{b}	5.41±0.13^{a}	3.43±0.11^{c}	3.77±0.10^{bc}	3.95±0.27^{ab}	4.16±0.08^{a}
Val	4.89±0.06^{a}	4.61±0.08^{b}	4.68±0.10^{b}	4.32±0.03^{c}	4.57±0.18^{b}	4.43±0.04^{b}	4.53±0.10^{b}	5.14±0.13^{a}	4.83±0.03^{a}	4.64±0.20^{a}	3.88±0.04^{b}	4.51±0.24^{a}
Met	2.65±0.09^{a}	1.75±0.03^{c}	2.45±0.16^{a}	2.60±0.01^{a}	1.76±0.13^{c}	1.21±0.05^{d}	2.21±0.14^{b}	2.66±0.09^{a}	2.31±0.14^{b}	1.15±0.11^{c}	2.32±0.03^{b}	2.99±0.12^{a}
Cys	1.91±0.07^{a}	0.81±0.10^{c}	1.11±0.04^{b}	1.04±0.04^{b}	2.64±0.15^{a}	1.04±0.14^{b}	0.68±0.07^{c}	0.69±0.04^{c}	2.10±0.16^{a}	1.35±0.10^{b}	0.30±0.02^{c}	0.52±0.04^{c}
Ile	4.85±0.33^{a}	4.71±0.13^{a}	3.21±0.21^{b}	2.88±0.05^{b}	4.29±0.07^{a}	4.17±0.08^{a}	3.55±0.19^{b}	3.20±0.06^{c}	4.34±0.10^{b}	4.74±0.16^{a}	3.52±0.14^{c}	3.12±0.04^{d}
Leu	9.76±0.50^{a}	9.48±0.37^{a}	7.14±0.40^{b}	6.89±0.10^{b}	6.27±0.02^{b}	5.92±0.21^{c}	6.08±0.21^{bc}	6.74±0.06^{a}	6.49±0.10^{a}	6.19±0.22^{a}	4.21±0.14^{c}	5.70±0.17^{b}
Phe	5.31±0.11^{a}	5.27±0.23^{a}	4.70±0.27^{b}	4.57±0.10^{b}	4.70±0.08^{b}	4.99±0.06^{a}	4.46±0.10^{c}	5.13±0.12^{a}	4.46±0.15^{b}	5.01±0.08^{a}	4.30±0.08^{b}	4.99±0.16^{a}
Lys	1.88±0.02^{b}	1.57±0.04^{b}	4.37±0.27^{a}	4.15±0.05^{a}	3.21±0.07^{b}	2.59±0.04^{c}	4.65±0.20^{a}	4.65±0.20^{a}	4.96±0.06^{c}	4.80±0.08^{c}	5.34±0.05^{b}	6.10±0.15^{a}
Total[1]	40.34±0.80^{a}	36.86±0.47^{bc}	39.28±1.94^{ab}	36.36±0.28^{c}	36.11±0.92^{bc}	34.08±0.19^{c}	36.73±1.48^{b}	40.85±0.36^{a}	39.80±0.72^{a}	38.24±0.62^{b}	34.34±0.50^{c}	39.91±0.24^{a}

Dispensable AA												
Asp	6.55±0.25^{d}	7.51±0.16^{c}	8.61±0.47^{b}	14.62±0.16^{a}	6.80±0.29^{bc}	7.10±0.11^{b}	8.04±0.03^{a}	6.51±0.13^{c}	11.63±0.08^{a}	9.50±0.40^{b}	10.91±0.83^{a}	8.74±0.51^{b}
Glu	18.25±0.96^{b}	20.04±0.17^{a}	16.04±1.03^{c}	15.48±0.58^{c}	23.71±0.59^{b}	25.60±0.72^{a}	20.47±0.38^{c}	14.43±0.30^{d}	19.04±0.32^{bc}	21.29±0.81^{a}	20.58±1.24^{ab}	17.31±0.92^{c}
Ser	4.43±0.10^{b}	4.14±0.16^{b}	5.00±0.31^{a}	4.51±0.11^{b}	3.77±0.15^{b}	3.92±0.02^{b}	5.11±0.12^{a}	4.79±0.23^{a}	4.78±0.26^{c}	4.86±0.16^{c}	5.32±0.22^{b}	5.85±0.09^{a}
Gly	2.38±0.06^{c}	2.02±0.05^{d}	5.52±0.23^{a}	5.05±0.06^{b}	3.33±0.14^{c}	3.16±0.04^{c}	5.64±0.09^{a}	4.72±0.15^{b}	5.08±0.16^{c}	4.58±0.25^{c}	6.39±0.16^{a}	5.69±0.39^{b}
Arg	5.62±0.24^{b}	6.82±0.06^{a}	5.31±0.41^{b}	5.24±0.08^{b}	5.72±0.57^{a}	6.08±0.28^{a}	6.03±0.52^{a}	5.50±0.21^{a}	6.53±0.06^{c}	7.95±0.32^{a}	7.24±0.20^{b}	7.23±0.13^{b}
Ala	7.56±0.08^{a}	7.44±0.10^{a}	6.04±0.44^{b}	5.89±0.07^{b}	3.38±0.13^{b}	3.30±0.07^{b}	4.39±0.20^{a}	4.23±0.33^{a}	4.02±0.06^{a}	3.38±0.17^{b}	3.50±0.14^{b}	3.67±0.39^{ab}
Pro	6.53±0.03^{ab}	6.90±0.05^{a}	6.42±0.33^{b}	5.82±0.07^{c}	9.08±0.44^{b}	10.19±0.11^{a}	9.16±0.40^{b}	10.46±0.24^{a}	3.24±0.30^{b}	3.65±0.12^{b}	4.59±0.11^{a}	4.77±0.24^{a}
Total[2]	51.33±0.83^{c}	54.88±0.50^{ab}	52.94±2.86^{bc}	56.60±0.47^{a}	55.79±0.09^{b}	59.36±0.93^{a}	58.85±1.38^{a}	50.64±0.66^{c}	54.32±0.14^{b}	55.20±1.41^{ab}	58.52±2.12^{a}	53.27±2.18^{b}
TAA[3]	91.67±0.28	91.74±0.76	92.22±4.80	92.96±0.25	91.91±0.99	93.44±0.92	95.58±2.76	91.49±0.86	94.12±0.81	93.44±1.59	92.86±1.74	93.19±0.2.09

[1] Total, combined of all indispensable amino acids.

[2] Total, combined of all dispensable amino acids.

[3] TAA, combined total of all amino acids.

[a, b, c] AA within a row in each kind of cereal with different superscript letters were significantly different ($p < 0.05$).

Values were means ± SD (n = 3).

Table S2
Amino acid score of raw grains from millet, highland barley and buckwheat.

Samples	Millet	Highland barley	Buckwheat	Reference pattern (mg /g protein)
Child (6 months to 3 years)				
Ile	1.52	1.34	1.36	32
Leu	1.48	0.95	0.98	66
Lys	0.33	0.56	0.87	57
Thr	1.09	0.93	1.50	31
Val	1.14	1.06	1.12	43
His	1.03	0.87	1.11	20
Sulfur AA	1.69	1.63	1.63	27
Aromatic AA	1.72	1.68	1.52	52
Limiting AA[1]	Lys	Lys	Lys	
Older child, adolescent, adult				
Ile	1.62	1.43	1.45	30
Leu	1.60	1.03	1.06	61
Lys	0.39	0.67	1.03	48
Thr	1.35	1.16	1.86	25
Val	1.22	1.14	1.21	40
His	1.28	1.09	1.38	16
Sulfur AA	1.98	1.92	1.92	23
Aromatic AA	2.19	2.13	1.93	41
Limiting AA[1]	Lys	Lys	N	

[1]Indicated the first limiting AA, the first limiting AA was the one with the lowest AAS value and all AAS values were higher than or equal to 100 indicated that no AA was limiting.
Values were means ± SD (n = 3).
